# Integrative bioinformatic analysis identifies differentially expressed gene targets as potential biomarkers for anaplastic thyroid cancer

**DOI:** 10.1186/s43046-025-00282-2

**Published:** 2025-05-12

**Authors:** Angel Sebastian Treviño-Juarez, Jose Gerardo Gonzalez-Gonzalez, Rene Rodriguez-Gutierrez, Adriana Sanchez-Garcia, Camilo Daniel Gonzalez-Velazquez

**Affiliations:** 1 Endocrinology Division, Department of Internal Medicine, University Hospital “Dr. José E. González”, Universidad Autónoma de Nuevo León, Monterrey, México; 2 Plataforma INVEST-Medicina UANL KER Unit, Universidad Autónoma de Nuevo León, Monterrey, 64460 México; 3 Thyroid, Head and Neck Cancer (THANC) Foundation, 10 Union Square East, Suite 5B, New York, NY 10003 USA

**Keywords:** Anaplastic thyroid cancer, Biomarkers, Cell division, Bioinformatics analysis, Differentially expressed genes

## Abstract

**Background:**

Anaplastic thyroid carcinoma (ATC) is among the most lethal thyroid malignancies, with poor clinical outcomes and limited treatment strategies. To gain insights into the molecular mechanisms involved in its progression, we performed an integrative bioinformatic analysis.

**Methods:**

We analyzed five microarray datasets from the GEO database to compare gene expression profiles between ATC samples and normal thyroid tissues. Differentially expressed genes (DEGs) were identified using GEO2R, and overlapping genes across datasets were detected through Venn diagram analysis. Functional enrichment was performed using DAVID and Metascape. A protein–protein interaction (PPI) network was constructed with STRING, and significant gene modules were identified using the MCODE plugin in Cytoscape. Co-expression analysis was further explored with GeneMANIA.

**Results:**

We identified 7532 DEGs, of which 3509 were upregulated and 4023 were downregulated. Upregulated genes were mainly involved in cell division and mitotic control, while downregulated genes were related to thyroid hormone production and gland development. Six hub genes stood out for their centrality in the network: TPX2, MAD2L1, CDC20, CDKN3, CENPF, and NEK2.

**Conclusion:**

Our findings shed light on key genes and pathways that may contribute to ATC pathogenesis. These results provide a foundation for identifying potential diagnostic biomarkers and therapeutic targets for this aggressive cancer.

## Background

Thyroid carcinoma (THCA) encompasses a diverse group of neoplasms, ranging from well-differentiated tumors to poorly differentiated and undifferentiated forms, such as anaplastic thyroid carcinoma (ATC) [1]. ATC, although rare, is among the most aggressive subtypes and has shown a gradual increase in incidence worldwide [2]. Despite accounting for only 2% of all thyroid cancers, it is responsible for approximately 13% of thyroid cancer-related deaths due to its highly lethal nature [3, 4]. The unfavorable prognosis of ATC is attributed to features such as marked cellular atypia, elevated mitotic index, and limited responsiveness to therapies typically used for differentiated thyroid carcinomas. Additionally, delayed diagnosis is common, often coinciding with extensive local invasion or distant metastasis, complicating clinical management. The aggressive behavior of ATC may stem from various genetic and structural abnormalities within tumor cells [5], highlighting the importance of elucidating its molecular underpinnings to develop effective therapeutic strategies [6]. In this context, bioinformatic approaches applied to gene expression data offer valuable tools for uncovering dysregulated pathways and genes involved in ATC. In the present study, we conducted an integrative analysis using publicly available GEO microarray datasets to identify differentially expressed genes (DEGs) in ATC. We further analyzed enriched Gene Ontology (GO) terms—covering biological processes (BP), cellular components (CC), and molecular functions (MF)—and performed KEGG pathway enrichment analysis. In addition, we constructed a protein–protein interaction (PPI) network to identify key hub genes potentially involved in ATC pathogenesis.

## Materials and methods

### Dataset selection from the GEO database

Gene expression datasets were retrieved from the Gene Expression Omnibus (GEO) repository (https://www.ncbi.nlm.nih.gov/geo/) based on predefined inclusion criteria. Studies were eligible if they (1) involved human samples (Homo sapiens), (2) utilized expression profiling by array, (3) were published between 2010 and 2022, and (4) included a minimum of three ATC tissue samples and three normal thyroid controls. Datasets lacking gene annotations, such as gene names or symbols, or without an associated peer-reviewed publication, were excluded to ensure data reliability and reproducibility.

### Identification of differentially expressed genes (DEG)

To determine DEGs between ATC and normal thyroid tissues, we used the GEO2R online tool (https://www.ncbi.nlm.nih.gov/geo/geo2r/), which enables comparison between sample groups within GEO datasets. Genes were considered differentially expressed if they met the following thresholds: |log2 fold change|≥ 1.0 and a *p*-value < 0.05. This filtering allowed us to focus on genes with statistically and biologically meaningful expression differences.

### Identification of overlapping upregulated and downregulated DEGs

To identify genes consistently upregulated or downregulated across datasets, we employed the jvenn web-based Venn diagram tool (http://jvenn.toulouse.inra.fr/app/example.html) [7]. This allowed us to visualize shared DEGs among the five selected studies and highlight genes with concordant expression patterns.

### Functional enrichment analysis (GO and KEGG pathway)

The functional relevance of the overlapping DEGs and identified hub genes was examined using two annotation tools: Metascape (http://metascape.org/gp/index.html#/main/step1) [8] and the Database for Annotation, Visualization and Integrated Discovery (DAVID) (https://david.ncifcrf.gov/) [9, 10]

We explored enrichment within the three major Gene Ontology (GO) categories: biological processes (BP), cellular components (CC), and molecular functions (MF), as well as KEGG pathways. Enrichment was considered significant at *p*-values < 0.05.

### PPI network and analysis of the module

To investigate interactions among DEGs, we built a protein–protein interaction (PPI) network using the STRING database (https://string-db.org/), applying a minimum confidence score of 0.4. The resulting network was analyzed using the MCODE plugin within Cytoscape to identify densely connected gene modules. Modules were selected based on the following criteria: MCODE score > 2, node score cutoff > 0.2, *k*-core = 2, and maximum depth = 100. Functional enrichment of these modules was then assessed to determine their biological significance. To further explore functional associations, co-expression analysis of hub genes was performed using the GeneMANIA platform (https://genemania.org/), allowing us to identify genes that share similar expression profiles and potential functional roles.

## Results

### Dataset selection from the GEO database

A total of five microarray datasets were selected for analysis: GSE27155, GSE65144, GSE85457, GSE53072, and GSE33630. These datasets included a combined total of 34 anaplastic thyroid carcinoma (ATC) tissue samples and 31 normal thyroid controls. Across all datasets, a total of 7532 differentially expressed genes (DEGs) were identified. Specifically, GSE27155 revealed 211 DEGs (76 upregulated, 135 downregulated), GSE65144 yielded 2166 DEGs (1344 upregulated, 822 downregulated), GSE85457 produced 2730 DEGs (1277 upregulated, 1453 downregulated), GSE53072 identified 875 DEGs (320 upregulated, 555 downregulated), and GSE33630 reported 1550 DEGs (492 upregulated, 1058 downregulated).

### Identification of overlapping up-regulated and down-regulated DEGs

**Fig. 1:**
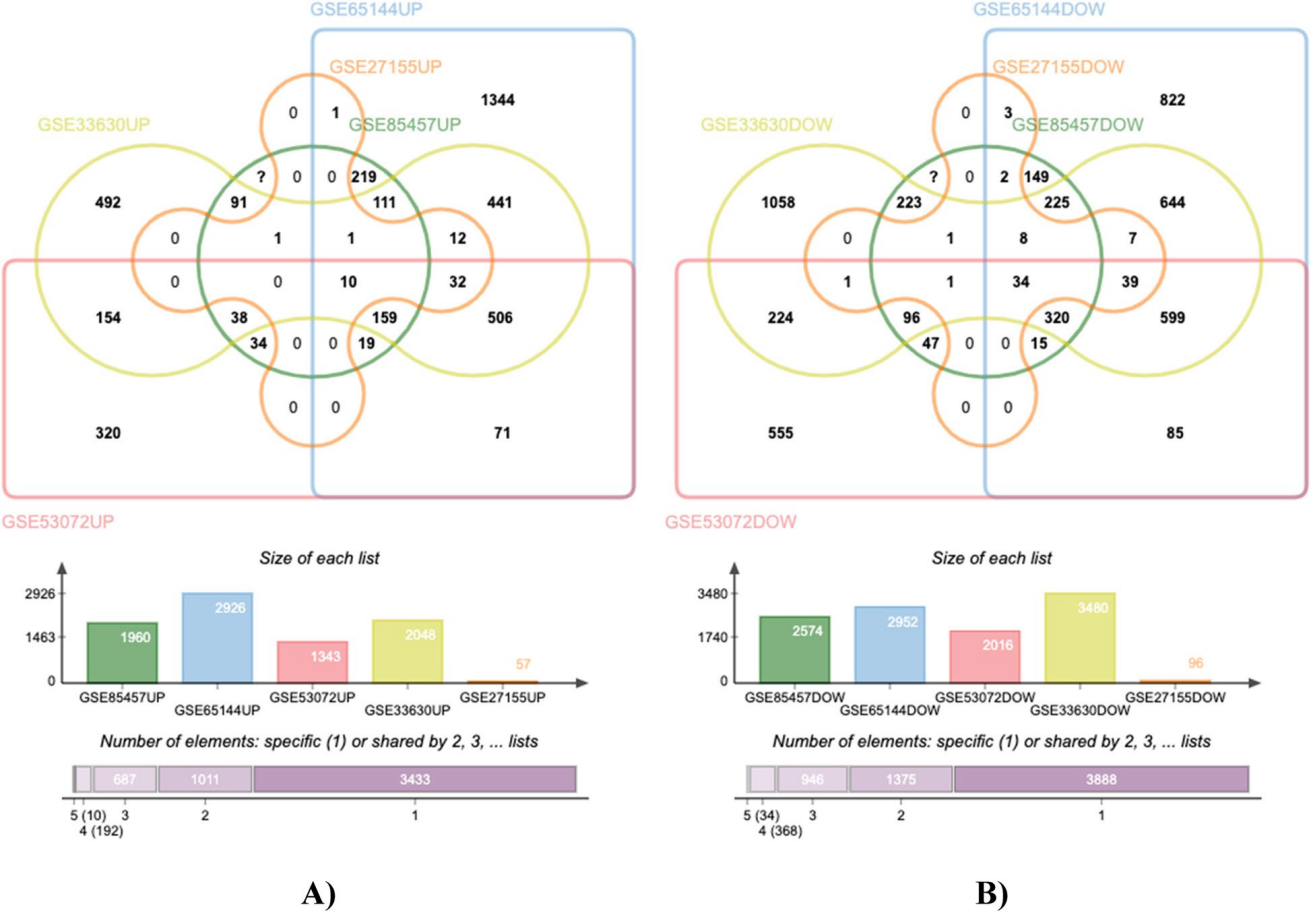
Venn diagrams of the overlapping DEGs. **A** Upregulated DEGs analysis. **B** Downregulated analysis

Cross-comparison using the jvenn tool revealed 44 genes consistently differentially expressed across the five datasets. Among these, 10 were upregulated and 34 were downregulated (Fig. 1). The upregulated DEGs included MAD2L1, TNFRSF21, NEK2, SCD, CDC20, CD163, CDKN3, CENPF, TPX2, and TLR2, while the downregulated DEGs comprised FOXE1, SORBS2, NKX2-1, NTRK2, DIO1, KCNJ16, SLC4A4, HOPX, MPPED2, CRYAB, CKB, ABCA8, DAPK2, HGD, ID4, FOS, PAX8, GPRASP1, SELENBP1, FCGBP, NEBL, KCNQ1, NR3C2, RAP1GAP, LMOD1, FGFR2, DUOX1, CD24, TG, CLIC3, TPO, FAM189A2, PCP4, and KCNJ15.

### Functional enrichment analysis (GO and KEGG pathway)

Among the upregulated DEGs, enrichment analysis revealed predominant involvement in biological processes (BP) such as cell division, mitotic spindle assembly checkpoint, and mitotic spindle organization. In terms of cellular component (CC), these genes were enriched in structures like the spindle pole, spindle, and mitotic checkpoint complex. For molecular function (MF), they were associated with protein C-terminus binding and general protein binding. KEGG pathway analysis linked these genes primarily to cell cycle regulation and oocyte meiosis (Table 1).

**Table 1:** Functional and pathway enrichment analysis of the up-regulated overlapping DEGs in ATC

Category	Term	Count	%	*P*-value
GOTERM BP	Cell division	5	50.0	1.8E − 5
GOTERM BP	Mitotic spindle assembly checkpoint	3	30.0	9.6E − 5
GOTERM BP	Mitotic spindle assembly	3	30.0	1.6E − 4
GOTERM CC	Spindle pole	5	50.0	1.9E − 7
GOTERM CC	Mitotic checkpoint complex	2	20.0	1.8E − 3
GOTERM CC	Spindle	3	30.0	1.8E − 3
GOTERM MF	Protein C-terminus binding	3	30.0	4.2E − 3
GOTERM MF	Protein binding	10	100.0	2.6E − 2
KEGG PATHWAY	Cell cycle	2	20.0	6.0E − 2
KEGG PATHWAY	Oocyte meiosis	2	20.0	6.3E − 2

*DEGs* Differentially expressed genes, *ATC* Anaplastic thyroid cancer, *GOTERM* Gene Ontology Term, *BP* Biological process, *CC* Cellular component, *MF* Molecular function, *KEGG* Kyoto Encyclopedia of Genes and Genomes

**Fig. 2:**
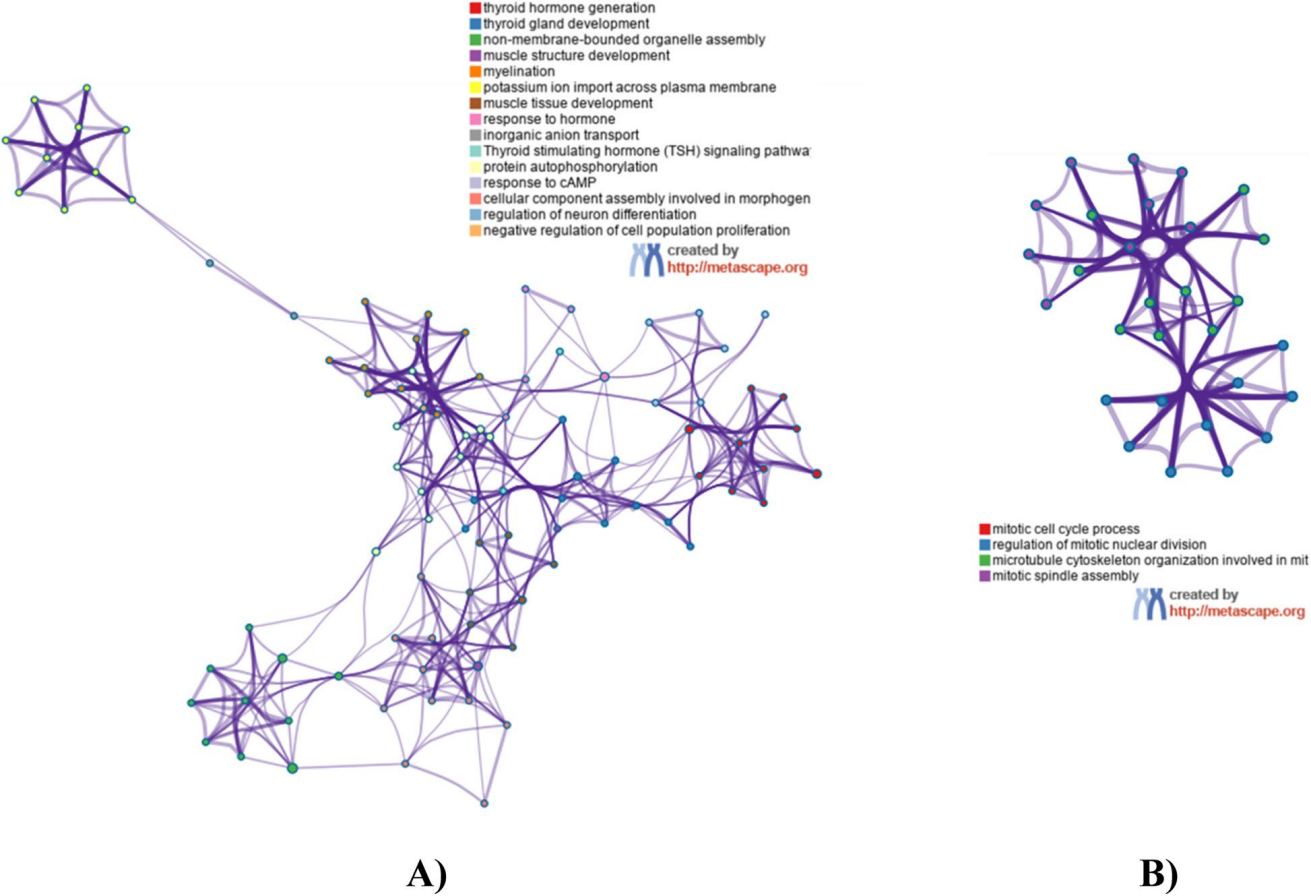
**A** Functional enrichment analysis of the overlapping DEGs using METASCAPE. **B** Functional enrichment analysis of the hub genes using METASCAPE

Conversely, the downregulated DEGs were significantly enriched in BP terms related to thyroid hormone production, biosynthesis of hormones, and thyroid gland development. Enrichment in CC terms pointed to cellular locations such as the membrane, cell surface, and basolateral plasma membrane. In the MF category, the downregulated genes were involved in transcription factor activity, sequence-specific DNA binding, selenium binding, and protein binding. KEGG analysis associated these genes with thyroid hormone synthesis, gastric acid secretion, and tyrosine metabolism pathways (Table 2). Additional functional analysis using Metascape is presented in Fig. 2.

**Table 2:** Functional and pathway enrichment analysis of the downregulated overlapping DEGs in ATC

Category	Term	Count	%	*P*-value
GOTERM BP	Thyroid hormone generation	5	14.7	6.5E−9
GOTERM BP	Hormone biosynthetic process	4	11.8	7.4E−7
GOTERM BP	Thyroid gland development	4	11.8	1.1E−5
GOTERM CC	Membrane	13	38.2	2.7E−4
GOTERM CC	Cell surface	7	20.6	4.1E−4
GOTERM CC	Basolateral plasma membrane	4	11.8	6.0E−3
GOTERM MF	Transcription factor activity, sequence-specific DNA binding	5	14.7	1.4E−2
GOTERM MF	Selenium binding	2	5.9	1.5E−2
KEGG PATHWAY	Protein binding	28	82.4	2.1E−2
KEGG PATHWAY	Thyroid hormone synthesis	4	11.8	8.8E−4

*DEGs* Differentially expressed genes, *ATC* Anaplastic thyroid cancer, *GOTERM* Gene Ontology Term, *BP* Biological process, *CC* Cellular component, *MF* Molecular function, *KEGG* Kyoto Encyclopedia of Genes and Genomes

*DEGs* differentially expressed genes, *ATC* anaplastic thyroid cancer, *GOTERM* Gene Ontology Term, *BP* biological process, *CC* cellular component, *MF* molecular function, *KEGG* Kyoto Encyclopedia of Genes and Genomes.

### PPI network and analysis of the module

**Fig. 3:**
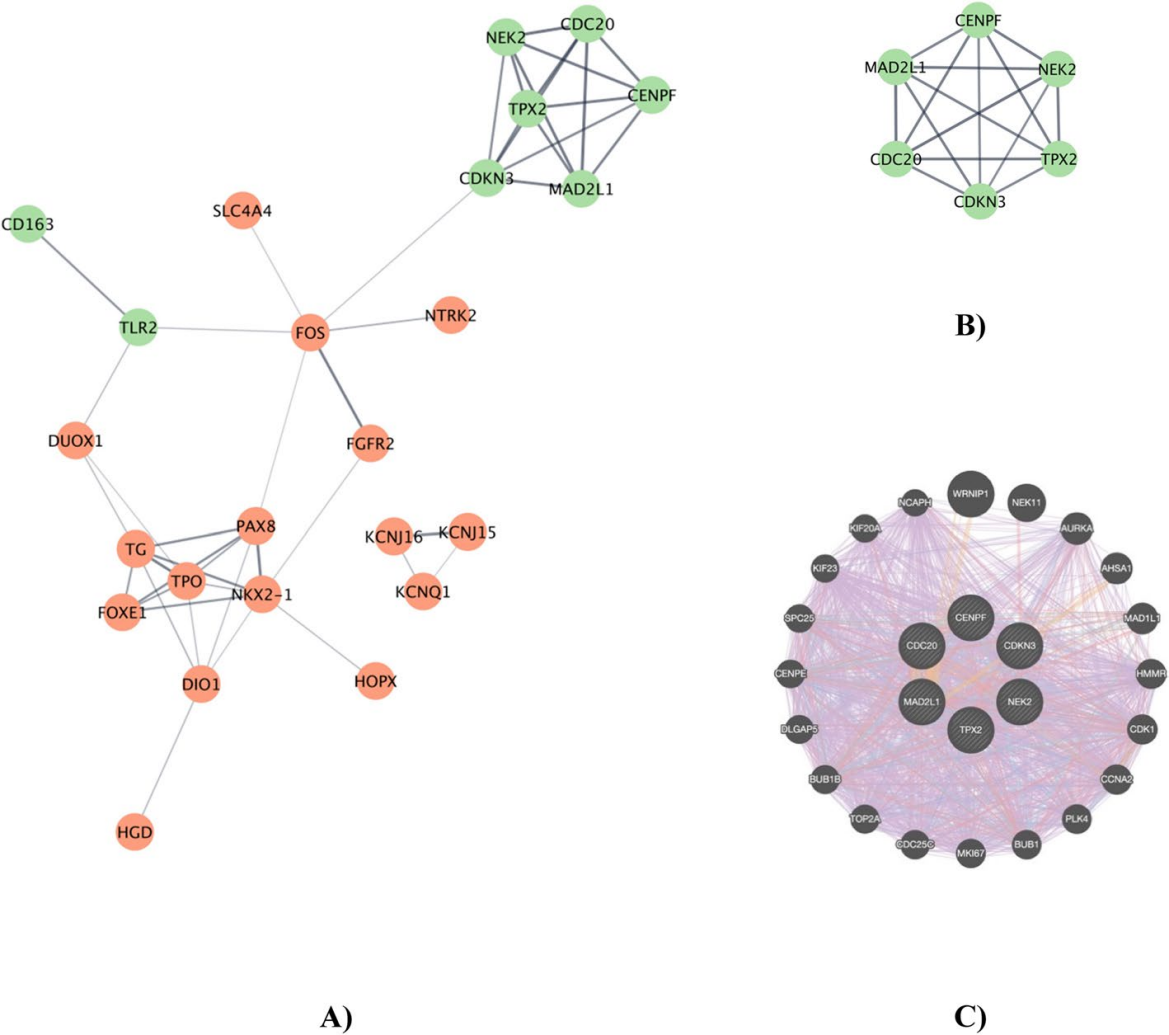
**A** Overlapping DEGs Protein-protein interaction network analysis by STRING platform (green nodle represents up-regulated DEG and red nodle represents down-regulated DEG). **B** Hub-genes identification analysis by MCODE using Cytoscape software. **C** Co-expression network analysis of the hub genes in ATC using GeneMANIA

The protein–protein interaction (PPI) network, constructed using STRING and visualized in Cytoscape (Fig. 3), consisted of 44 nodes and 45 edges, corresponding to the 10 upregulated and 34 downregulated overlapping DEGs. Using the MCODE plugin, six hub genes were identified: TPX2, MAD2L1, CDC20, CDKN3, CENPF, and NEK2.

GO enrichment of these hub genes revealed strong associations with biological processes such as cell division, mitotic checkpoint regulation, and spindle assembly. They were also enriched in cellular structures like the spindle pole and perinuclear cytoplasm, and functions including protein C-terminus and microtubule binding. KEGG pathway analysis showed significant enrichment in cell cycle pathways, oocyte meiosis, and human T-cell leukemia virus 1 infection (Table 3). The co-expression relationships of these hub genes are depicted in the GeneMANIA network shown in Fig. 3, alongside functional enrichment results from Metascape.

**Table 3:** Hub-genes enrichment and pathway analysis

Category	Term	Count	%	*P*-value
GOTERM BP	Cell division	5 (NEK2, TPX2, CDC20, CENPF, MAD2L1)	83.3	7.5E−7
GOTERM BP	Mitotic spindle assembly checkpoint	3 (CDC20, CENPF, MAD2L1)	50.0	2.7E−5
GOTERM BP	Mitotic spindle assembly	3 (NEK2, TPX2, CDC20)	50.0	4.4E−5
GOTERM CC	Spindle pole	5 (NEK2, TPX2, CDC20, CENPF, MAD2L1)	83.3	7.5E−9
GOTERM CC	Perinuclear region of cytoplasm	4 (CDC20, CENPF, CDKN3, MAD2L1)	66.7	4.9E−4
GOTERM CC	Spindle	3 (TPX2, CDC20, CENPF)	50.0	5.1E−4
GOTERM MF	Protein C-terminus binding	3 (CDC20, CENPF, MAD2L1)	50.0	1.2E−3
GOTERM MF	Microtubule binding	2 (TPX2, CENPF)	33.3	6.9E−2
KEGG PATHWAY	Cell cycle	2 (CDC20, MAD2L1)	33.3	1.5E−2
KEGG PATHWAY	Occyte meiosis	2 (CDC20, MAD2L1)	33.3	1.6E−2
KEGG PATHWAY	Human T-cell leukemia virus 1 infection	2 (CDC20, MAD2L1)	33.3	2.7E−2

*DEGs* Differentially expressed genes, *GOTERM* Gene Ontology Term, *BP* Biological process, *CC* Cellular component, *MF* Molecular function, *KEGG* Kyoto Encyclopedia of Genes and Genomes

## Discussion

In this study, we performed an integrative bioinformatics analysis of five gene expression datasets to investigate molecular alterations associated with anaplastic thyroid carcinoma (ATC). Our analysis identified 7532 differentially expressed genes (DEGs), including 3509 upregulated and 4023 downregulated genes. Functional enrichment highlighted the involvement of these DEGs in biological processes such as cell cycle regulation, mitosis, thyroid hormone biosynthesis, and gland development. These results align with existing literature emphasizing the role of disrupted mitotic control and impaired thyroid function in ATC pathogenesis, consistent with the tumor’s aggressive clinical behavior.

Through protein–protein interaction (PPI) network construction and module analysis, we identified six hub genes—TPX2, MAD2L1, CDC20, CDKN3, CENPF, and NEK2—that may play central roles in ATC progression. These genes are functionally connected to critical pathways involved in cell division and chromosomal integrity, making them promising targets for future experimental validation and therapeutic exploration.

TPX2 microtubule nucleation factor (TPX2), a microtubule nucleation factor, plays an essential role in mitotic spindle assembly and has been implicated in the proliferation of various cancer types, including thyroid carcinoma [11, 12]. Its downregulation has been associated with impaired mitotic progression and reduced tumor growth [13, 14]. While previous research has primarily explored its role in other malignancies such as gastric, breast, colon, liver, prostate, and endometrial cancers, our findings underscore its potential relevance in ATC, where it may act as a driver of uncontrolled proliferation [15–19]. Mitotic arrest deficient 2 like 1 (MAD2L1), a key regulator of the spindle assembly checkpoint, has been linked to genomic instability and increased proliferation in several solid tumors, including lung and breast cancers [20, 21]. Its altered expression has been associated with chromosomal missegregation and aneuploidy [22]. However, its specific contribution to ATC remains unclear, and further investigation is needed to understand its mechanistic role in this aggressive tumor type. Cell division cycle 20 (CDC20), a regulatory component of the anaphase-promoting complex (APC), is known to facilitate cell cycle progression and has been found to be overexpressed in multiple malignancies, such as breast, lung, colorectal, prostate, and hepatocellular carcinomas [23, 24]. Importantly, recent studies have validated its upregulation in ATC tissues and consistently identified it as a hub gene in transcriptomic analyses [25–27]. These findings highlight CDC20 as a strong candidate for targeted therapeutic development. Cyclin-dependent kinase inhibitor 3 (CDKN3), encodes a cyclin-dependent kinase inhibitor that regulates the G1/S transition in the cell cycle [28]. Although traditionally considered a tumor suppressor, its aberrant expression and splicing have been reported in various cancers, including ATC and poorly differentiated thyroid carcinomas [29]. Its involvement in cell cycle deregulation makes it a potential target for further therapeutic research in ATC. Centromere protein F (CENPF), a centromere-associated protein involved in chromosome segregation [30]. Has been recognized as a hub gene in ATC through previous bioinformatics studies [31–33]. High CENPF expression has been associated with worse overall survival, suggesting its potential role in driving tumor aggressiveness [32]. These data support its consideration as both a prognostic marker and a possible therapeutic target. NIMA-related kinase 2 (NEK2), a serine/threonine kinase involved in mitotic regulation and centrosome separation [34] Its overexpression has been extensively documented in a variety of cancers, including breast, non-small cell lung cancer, and myeloma, where it contributes to tumorigenesis through mechanisms such as chromosomal instability, centrosome amplification, and mitotic defects [35, 36]. In these malignancies, NEK2 is also strongly associated with poor survival outcomes, underscoring its role as a marker of poor prognosis. In addition to promoting tumorigenesis, NEK2 has been linked to drug resistance through the activation of efflux drug pumps, a mechanism that predicts rapid relapse and treatment failure in cancers like myeloma [36]. While its role in ATC has not been fully elucidated, our analysis suggests it may contribute to the invasive and treatment-resistant phenotype observed in this carcinoma.

This study integrates gene expression data from five independent datasets to provide a broader and more reliable understanding of the molecular landscape in anaplastic thyroid carcinoma (ATC). By identifying overlapping differentially expressed genes (DEGs) across multiple cohorts, we minimized dataset-specific biases and enhanced the robustness of our results. This integrative approach not only reinforces previously reported molecular alterations in ATC but also uncovers novel genes and pathways that may be involved in its pathogenesis. The six identified hub genes TPX2, MAD2L1, CDC20, CDKN3, CENPF, and NEK2 show potential as early diagnostic biomarkers or as targets for therapeutic development, offering a foundation for precision medicine strategies in this aggressive malignancy. Moreover, the use of publicly available datasets allows for reproducibility and transparency, and bioinformatic tools provide a cost-effective means to screen for key molecular players prior to initiating experimental work. These findings may support the development of targeted therapies and improve our understanding of the molecular mechanisms underlying ATC progression and treatment resistance. However, this study is not without limitations. The exclusive reliance on transcriptomic data from bulk tumor samples introduces potential variability due to sample heterogeneity, platform differences, and lack of cellular resolution. While integration across datasets helps mitigate some of these challenges, variability in clinical annotation and processing methods cannot be fully controlled. Additionally, the conclusions drawn are based on in silico analysis and require further experimental validation. Future research should focus on functional assays such as gene silencing or overexpression studies in ATC cell lines and animal models to confirm the roles of these hub genes. Integrating multi-omics data and correlating findings with clinical outcomes will be essential to translating these molecular insights into therapeutic advances.

## Conclusion

In conclusion, this study identified 7532 differentially expressed genes (DEGs) in anaplastic thyroid carcinoma (ATC), offering a deeper understanding of the molecular changes associated with its aggressive phenotype. By integrating data from five independent GEO datasets, we uncovered six hub genes TPX2, MAD2L1, CDC20, CDKN3, CENPF, and NEK2 which are consistently involved in essential pathways related to mitosis and cell cycle regulation. These genes emerge as promising candidates for early diagnosis and as potential targets for novel therapeutic strategies. The integrative approach adopted here reduces the limitations associated with single dataset studies and enhances the robustness of our findings. In addition to supporting previous observations, our analysis highlights novel molecular features of ATC that may guide future research and clinical applications. While these results enhance the understanding of ATC at the molecular level, future experimental validation will be essential to confirm their clinical relevance and advance the development of precision medicine strategies aimed at improving outcomes for ATC patients.

## Data Availability

Not applicable.

## Abbreviations

ATC: Anaplastic thyroid carcinoma
DEGs: Differentially expressed genes
PPI: Protein-protein interaction
MCODE: Molecular complex detection
THCA: Thyroid carcinoma
ATC: Anaplastic thyroid carcinoma
GO: Gene Ontology
BP: Enriched terms, including biological process
CC: Cellular component
MF: Molecular function
KEGG: Kyoto Encyclopedia of Genes and Genomes
GEO: Gene Expression Omnibus
STRING: Search Tool for Retrieval Interaction Genes
TPX2: TPX2 microtubule nucleation factor
SAC: Spindle assembly checkpoint
MAD2L1: Mitotic arrest deficient 2 like 1
CDC20: Cell division cycle 20
APC: Anaphase-promoting complex
CDKN3: Cyclin-dependent kinase inhibitor 3
CDKs: Cyclin-dependent kinases
CENPF: Centromere protein F
NEK2: NIMA-related kinase 2
